# An efficient algorithm for improving structure-based prediction of transcription factor binding sites

**DOI:** 10.1186/s12859-017-1755-0

**Published:** 2017-07-17

**Authors:** Alvin Farrel, Jun-tao Guo

**Affiliations:** Department of Bioinformatics and Genomics, University of North Carolina at Charlotte, 9201 University City Blvd, Charlotte, NC 28223 USA

**Keywords:** Transcription factor binding site, Structure-based prediction, Binding motif, Integrative energy function, Fragment-based method, Pentamer

## Abstract

**Background:**

Gene expression is regulated by transcription factors binding to specific target DNA sites. Understanding how and where transcription factors bind at genome scale represents an essential step toward our understanding of gene regulation networks. Previously we developed a structure-based method for prediction of transcription factor binding sites using an integrative energy function that combines a knowledge-based multibody potential and two atomic energy terms. While the method performs well, it is not computationally efficient due to the exponential increase in the number of binding sequences to be evaluated for longer binding sites. In this paper, we present an efficient pentamer algorithm by splitting DNA binding sequences into overlapping fragments along with a simplified integrative energy function for transcription factor binding site prediction.

**Results:**

A DNA binding sequence is split into overlapping pentamers (5 base pairs) for calculating transcription factor-pentamer interaction energy. To combine the results from overlapping pentamer scores, we developed two methods, Kmer-Sum and PWM (Position Weight Matrix) stacking, for full-length binding motif prediction. Our results show that both Kmer-Sum and PWM stacking in the new pentamer approach along with a simplified integrative energy function improved transcription factor binding site prediction accuracy and dramatically reduced computation time, especially for longer binding sites.

**Conclusion:**

Our new fragment-based pentamer algorithm and simplified energy function improve both efficiency and accuracy. To our knowledge, this is the first fragment-based method for structure-based transcription factor binding sites prediction.

**Electronic supplementary material:**

The online version of this article (doi:10.1186/s12859-017-1755-0) contains supplementary material, which is available to authorized users.

## Background

Transcription factors (TFs) interact with specific DNA sequences, called transcription factor binding sites (TFBSs), to regulate gene expression [1, 2]. Genome-wide TFBS identification, a crucial step in deciphering transcription regulatory networks and annotating genomic sequences, remain a key challenge in post-genomics research. Both high-throughput experimental methods and computational approaches have been developed to tackle this problem. Each method has its unique advantages and limitations [3].

**Fig. 1:**
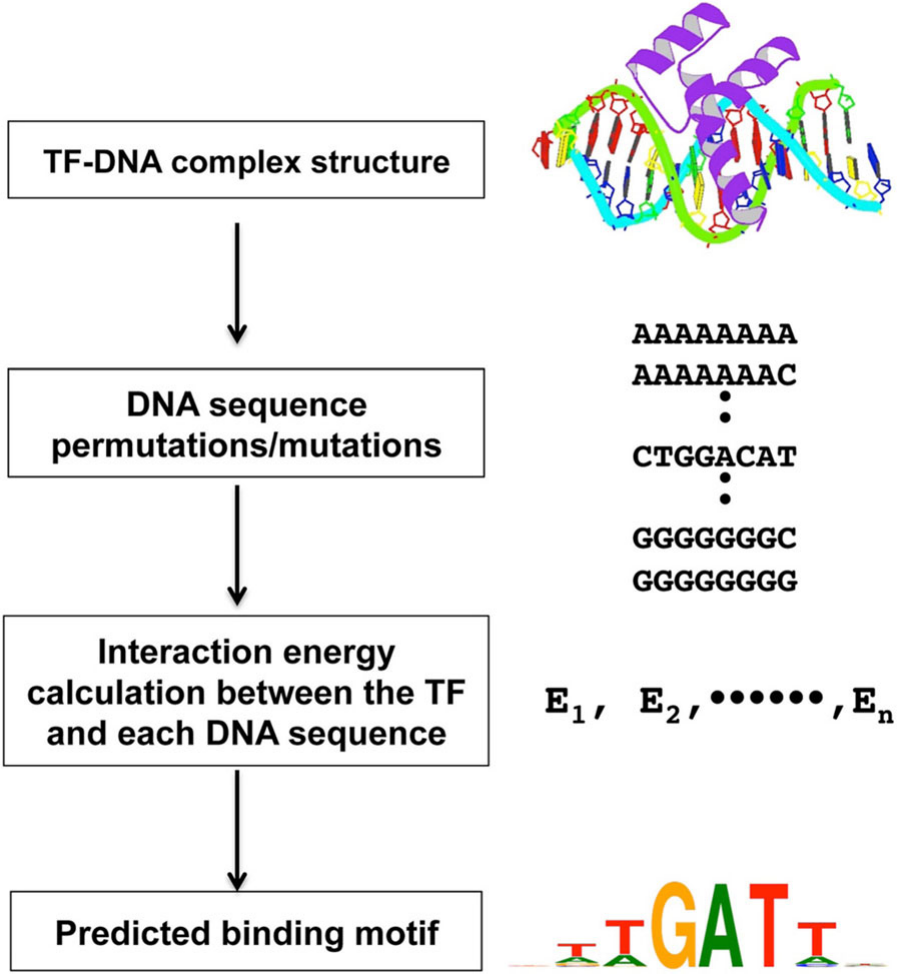
Four major steps in structure-based prediction of transcription factor binding sites

Computational methods include sequence-based and structure-based TFBS predictions. Structure-based prediction methods take advantage of the increasing numbers of TF-DNA complex structures in Protein Data Bank (PDB) [4, 5]. Unlike sequence-based methods that rely on sequence conservation and usually are family based, structure-based TFBS prediction methods consider the physical interactions between a TF and candidate binding sequences (Fig. 1). The advantage of structure-based TFBS prediction methods lies in that they can explain the possible mechanisms involved in specific TF-DNA binding and recognition, and help understand the effects of mutations on gene expression since these methods mimic in vivo binding and recognition events. A typical structure-based TFBS prediction method evaluates each candidate DNA sequence by “threading” it onto the DNA structure of a known TF-DNA complex and the binding affinity or binding energy is then calculated using energy functions [3]. Virtually all structure-based methods for TFBS prediction require a TF-DNA interaction model that can be experimentally solved protein-DNA complex structures [6–8] or high quality homologous TF-DNA models as it has been demonstrated that transcription factors from the same family, in general, interact with DNA in a similar manner [9–11]. One issue in structure-based TFBS prediction concerns the potential divergence of DNA structures of different sequences for a transcription factor since only one TF-DNA complex model is used for evaluating different sequences. For this method to work, TF-DNA binding modes and DNA structures should be very similar. Our recent survey showed that the DNA structures of different cognate binding sequences of a transcription factor generally conserve well with smaller root mean squared deviations (RMSDs), suggesting that the interaction modes are conserved between the transcription factor and its specific binding sequences even though there are variations at certain positions of the binding motif [3].

The binding affinities between TFs and their binding sequences are evaluated using an energy function, which can be knowledge-based, physics-based or a combination of both [12]. Knowledge-based energy functions are derived from statistical analysis of a set of known, non-redundant protein-DNA complexes. Their resolution varies from residue-level [13–16] to atom-level potentials [17–20]. Physics-based energy functions, on the other hand, perform atomic level physicochemical calculations to quantify electrostatic interactions, van der Waals (VDW) forces, solvation energy, and others [4]. Using physics-based energy can be computationally expensive and the method is sensitive to conformational changes. This is important since x-ray structures represent the majority of TF-DNA complexes in PDB, which are snapshots of dynamic ensembles of many possible conformations. Knowledge-based potentials, on the other hand, are less sensitive to conformational changes because of their relatively coarse-level and mean-force nature, and are more computationally efficient. However these potentials are “averaged” values among different types of interactions and are less accurate for some amino acid types due to low count problem [12].

To take advantage of the unique features of both knowledge-based and physics-based potentials for structure-based TFBS prediction, we recently developed an integrative energy (IE) function that consists of three terms, a residue-level knowledge-based multibody (MB) potential, an explicit hydrogen bond (HB) energy, and an electrostatic potential for π-interaction energy. Studies have shown that both hydrogen bonds and π-π interactions play critical roles in specific protein-DNA binding [12]. Even though the multibody potential implicitly captures biophysical interactions including hydrogen bonds and π-interactions, the mean-force nature and the typical low count problem limit its ability to capture the key hydrogen bonds and π-interactions that contribute to TF-DNA binding specificity [15]. The IE function improved TFBS prediction accuracy over MB as well as DDNA3, a knowledge-based atomic-level protein-DNA interaction potential [12, 15, 18]. However, the algorithm cannot scale well for prediction of longer TF binding sites, especially for binding sites from TF dimers or tetramers. As shown in Fig. 1, our previous prediction algorithm first generates TF-DNA complexes consisting of a TF and every possible permutation of its target sequence using 3DNA [21, 22]. The IE function is then applied to each TF-DNA complex to calculate their binding energy and subsequently predict their binding sites (Fig. 1) [12]. The total number of TF-DNA complex energy calculations is 4^L^, where L is the length of the binding motif. For example, in our previous approach, we used a binding sequence of length 8, which requires evaluating a total of 65,536 TF-DNA complexes. As the size of the binding sites increases, the time complexity increases exponentially.

Here we propose a new approach, called pentamer algorithm, which splits the DNA binding sequence into a series of overlapping subsequences/fragments of length 5 base pairs (bps), for more efficient and accurate TFBS prediction. Fragment-based methods have shown their power in the field of protein structure prediction [23, 24]. Also in DNA shape studies, Rohs et al. have developed a DNA pentamer model for predicting DNA structural features [25–28]. In their model, DNA shape features of a nucleotide are predicted from a pentamer sequence that takes sequence context, two on each side, into consideration [28]. Even though we used the same term “pentamer” for DNA fragments in this work, the research problems are different. One uses a pentamer model or a 5-bp sliding window to predict the shape features of the center nucleotide [25–28]. Our approach, on the other hand, calculates TF-DNA pentamer binding energy and the full-length binding sites are predicted by combining the fragment scores in post-preocessing. To the best of our knowledge, our method is the first attempt to use DNA fragments for structure- or interaction-based TF binding site prediction. In addition to the pentamer algorithm, we modified our IE function to simplify the calculation of the hydrogen bond energy and π-interaction energy proposed in our previous method [12]. Results show that our new approach not only dramatically improve the prediction speed, it also helps improve prediction accuracy, especially for TF dimers with longer binding sites.

## Methods

### Modified integrative energy function

In our previous study, the IE function consisted of a multibody potential, a hydrogen bond term and a π-interaction term [12]. The knowledge-based multibody potential utilizes structural environment for accurate assessment of protein-DNA interactions as it uses DNA tri-nucleotides, called triplets, as an interaction unit to study interactions between TF and DNA [15]. The potential is distance dependent and the distance is calculated between an amino acid’s β-carbon and the geometric center of a nucleotide triplet. The position of a nucleotide is represented by the N1 atom in pyrimidines or the N9 atom in purines [15]. The hydrogen bond energy was calculated using FIRST, a third party program [12, 29], which makes the calculation less efficient. Since electrostatic interactions are involved in both hydrogen bond and π-interactions, in this study, we combine the hydrogen bond energy and π-interaction energy into one electrostatic energy term to reduce the complexity of energy calculation. The modified integrative energy function is shown in Eq. 1.

1 $$ {E}_{IE}={W}_{MB}{E}_{MB}+{W}_E{E}_E $$

where *E*_*IE*_ is the new, simplified integrative energy score, *W*_*MB*_ and *E*_*MB*_ are the weight and normalized energy score for the multibody potential respectively, and *W*_*E*_ and *E*_*E*_ are the weight and normalized score for the electrostatic energy respectively. Each energy term is normalized using the Min-Max normalization method as we described in our previous study [12]. Since there are only a limited number of non-redundant TF-DNA complexes with known TFBSs, not enough to have a separate training set for weight optimization, we used weights of 1 and 0.5 for *W*_*MB*_ and *W*_*E*_ respectively. The electrostatic term has smaller weight than *W*_*MB*_ since electrostatic interactions are already implicitly captured in the multibody potential. The electrostatic potential is calculated using a variation of coulombs law (Eq. 2) where the partial charges of the atoms within interaction distance were determined using Marvinsketch, from Chemaxon (Additional file 1: Table S1) [30].

2 $$ {E}_{a b}=\frac{k_e{N}_A{q}_a{q}_b}{\varepsilon d} $$

where *E*_*ab*_ is the electrostatic energy between an atom *a* of an amino acid and an atom *b* of a DNA base, *k*_*e*_ is Coulomb’s constant. *N*_*A*_ is Avogadro’s number, *q*_*a*_ and *q*_*b*_ are the charges of the two atoms. *ε* is the dielectric constant and *d* is the distance between the point charges. The charges, *q*_*a*_ and *q*_*b*_, are determined by multiplying the partial charge values with the charge of an electron (1.6 × 10^−19^ coulombs). The electrostatic potential of each atomic interaction is added together for the total electrostatic energy between the TF and a specific DNA sequence as shown in Eq. 3.

3 $$ {E}_E=\sum_{N_{ab}}{E}_{ab} $$

where *E*_*E*_ is the total electrostatic energy between the TF and a DNA binding site, *N*_*ab*_ is the number of amino acid-base interactions, *E*_*ab*_ is the electrostatic energy between atom *a* of an amino acid and atom *b* of a base. The interaction distance *d* for atoms involved in hydrogen bond interaction was set at between 1.5 Å and 2.9 Å, a typical distance between the hydrogen atom and the hydrogen bond acceptor atom [21, 31–33]. We used REDUCE to add hydrogen atoms to the TF-DNA complex structures [34]. The cutoff distance for atoms involved in a possible π-interaction between an aromatic amino acid and a base was 4.5 Å based on previous studies [35]. The sum of the charges found in the electron cloud of aromatic residues, were used as the charge for the electrostatic energy calculation to account for the delocalization of electrons in π-systems and their involvement in π-π interaction [12, 36–38].

We have demonstrated that the original IE function outperforms both residue- and atomic-level knowledge-based potentials in structure-based prediction of TF binding sites [12], therefore, in this study we only compared prediction accuracy of our new pentamer algorithm (with a simplified IE function) to the original IE function.

### Pentamer algorithm

#### Generation of TF-pentamer DNA complexes

**Fig. 2:**
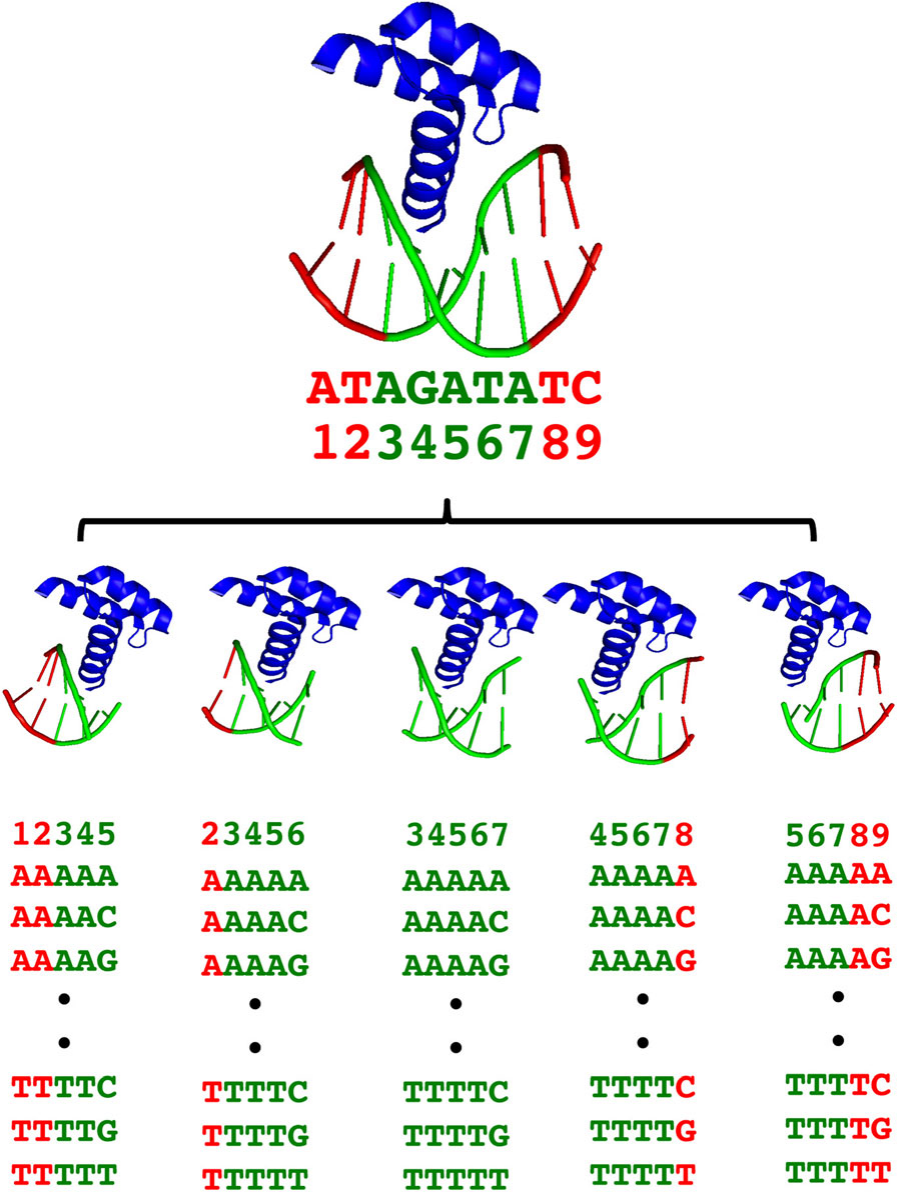
The DNA sequence is split into overlapping fragments of 5 bps. The green bases are TF-DNA contact sequences of length *n* = 5 bp and the red bases are the 2 flanking bases on each side. The number of TF-pentamer complexes to be evaluated is 5*1024 = 5120

The first step of the algorithm is to determine the binding sequence for a transcription factor. It can be based on prior knowledge or automatically detected using the TF-DNA complex structure. For automatic detection, the TF-DNA complex is checked for the first and the last base that are in contact with the TF using a distance cutoff of 5 Å between heavy atoms. Though the non-interacting flanking base pairs are less conserved, recent studies have shown that these flanking bases contribute to DNA binding specificity by affecting DNA shape and stability [27, 39–43]. Therefore we added two bases on each side of the binding sequence of length *n,* which resulted in an *n* + 4 DNA sequence for the initial system (Fig. 2). For example, a DNA binding sequence of 5 base pairs becomes a 9 bp sequence after adding two flanking base pairs on each side (Fig. 2). Energy minimization was first performed on the TF-DNA complex using UCSF Chimera 1.8 with the following parameters: 100 steepest descent steps with a step size of 0.02, 100 conjugate gradient steps with a step size of 0.02, and an update interval of 10 as described in our previous study [12, 33]. The DNA sequence was then split into a series of overlapping 5 bp sequences by shifting one base pair at a time. The DNA sequence in each TF-pentamer was mutated to every possible permutation using 3DNA [21, 22], which resulted 4^5^ or 1024 TF-pentamer complex structures for each original TF-pentamer. In total, there are n*1024 TF-pentamer complex structures to be evaluated, where *n* is the number of pentamer fragments from the original DNA structure. The binding energy for each TF-pentamer DNA complex was then calculated (Eq. 1).

#### Binding motif prediction

**Fig. 3:**
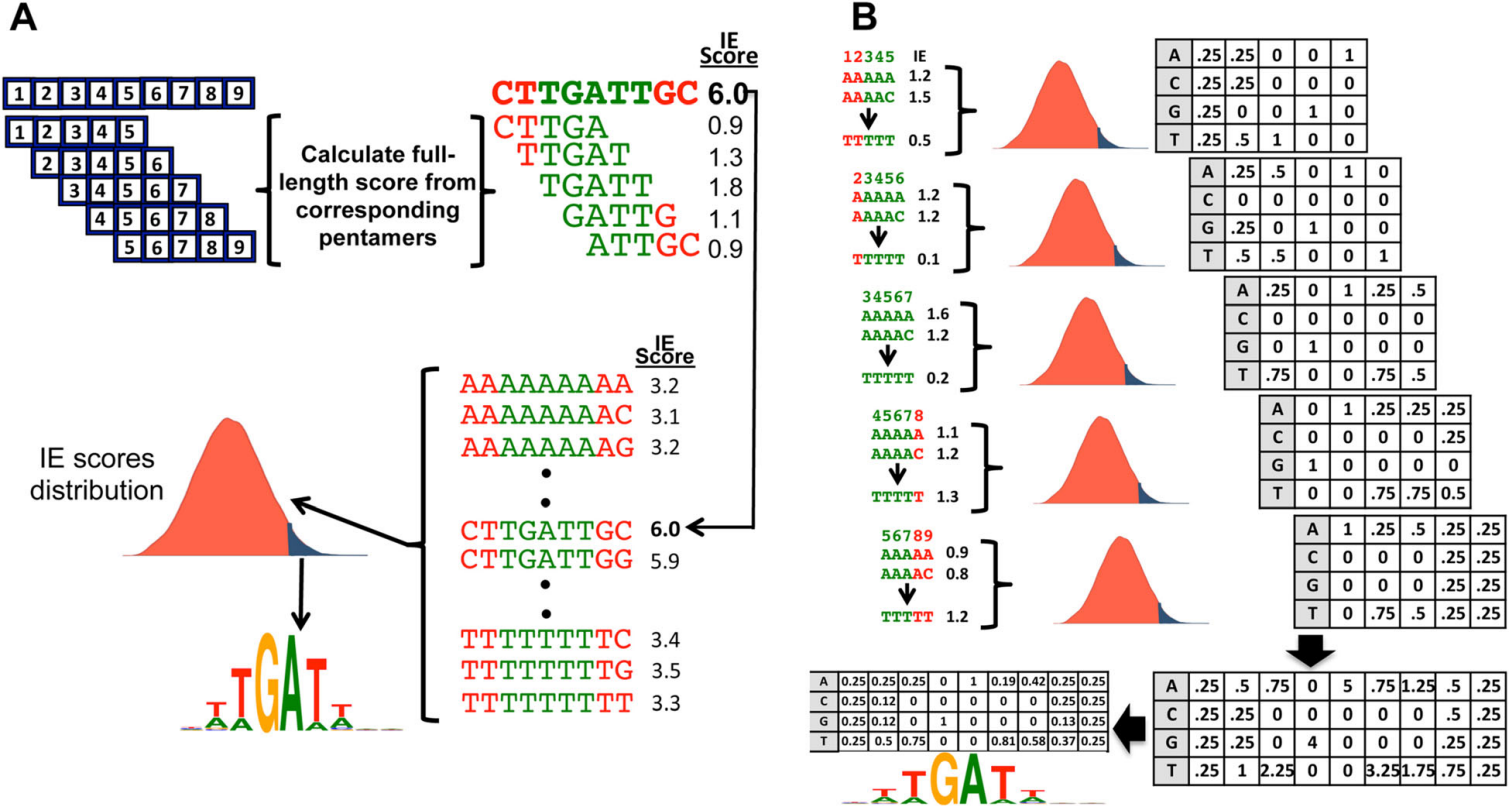
Algorithms for binding motif prediction based on TF-pentamer interaction energies. **a** In the Kmer-Sum algorithm, the IE score between a TF and a full-length DNA sequence is a summation of all the IE scores of the TF-pentamer complex (*top part*). After all IE scores between the TF and each permutation of the full-length DNA sequence are calculated, the binding motif is predicted based on the sequences with statistically significant IE scores among all possible sequences (*lower part*). **b** The PWM stacking algorithm generates a distribution of IE scores based on the sequence permutations for each pentamer of the original sequence. The PWM positions corresponding to the same position in the original structure are added together to form a PFM representing the TF’s TFBS, which is then converted to a PWM and binding motif logo

To predict the TF binding motif from these TF-pentamer interaction energies, we developed two different methods, Kmer-Sum algorithm and position weight matrix (PWM) stacking algorithm (Fig. 3). In the Kmer-Sum algorithm, the IE score of a full-length binding sequence is the sum of the interaction energy of overlapping pentamer sequences with the TF and the score of each permutation of the full-length binding sequence is calculated accordingly (Fig. 3a). The statistically significant scores from the binding sequence IE score distribution of all the full-length permutations were determined and their corresponding DNA sequences were used to generate a binding motif as described previously (Fig. 3a) [12]. In this study, the critical value for statistical significance in the Kmer-Sum algorithm was 0.01 normalized by the length of the predicted motif. For the PWM stacking algorithm (Fig. 3b), the binding sequence was broken up into pentamer subsequences. The IE score of each permutation of each pentamer sequence was calculated. For a given pentamer representing 5 contiguous bases of the binding motif, a PWM representing the statistically significant pentamer sequences was calculated from the distribution of IE scores of all possible pentamer permutations (Fig. 3b). Each position (column) in a pentamer PWM represents a specific position (column) in the binding motif PWM. All of the corresponding cells representing the frequency of a particular nucleotide in a specific position were added together to generate a position frequency matrix (PFM) of the binding motif (Fig. 3b). The PFM was then converted to a PWM and a motif logo using the method described by Schneider and Stephens [44, 45].

The TF-pentamer energy calculation and binding motif prediction were run on a cluster (a total of 708 computing cores) with dual Intel Xeon 2.93 GHz 6-core processors–X5670 and 3GBs RAM per core. The CPU time was recorded and the speed was compared with the full-length prediction method using IE, MB and DDNA3 energy fucntions.

### Dataset

The new method was tested on two non-redundant sets: TF monomer-DNA complexes and TF dimer-DNA complexes [12]. The dataset consisted of high quality X-ray crystal structures of TF-DNA complexes (resolution <3 Å, and R-factor ≤ 0.3) in PDB with corresponding JASPAR PWMs [46]. All TF monomer chains share no more than 35% sequence identity. The TF monomer dataset contains 27 non-redundant TF chain-DNA complexes representing 12 transcription factor families: helix loop helix, zinc fingers, homeodomains, leucine zippers, STAT1, fork head, ETS family, high mobility group (HMG), NFAT, SMAD, P53 DNA binding domain, and runt domains. The dataset include: 1 AM9:A, 1 BC8:C, 1BF5:A, 1DSZ:A, 1GU4:A, 1H9D:A, 1JNM:A, 1LLM:C, 1NKP:A, 1NKP:B, 1NLW:A, 1OZJ:A, 1P7H:L, 1PUF:A, 1PUF:B, 2A07:F, 2 AC0:A, 2DRP:A, 2QL2:A, 2QL2:B, 2UZK:A, 2YPA:B, 3F27:A, 3HDD:A, 4F6M:A, 4HN5:A, 4IQR:A.

For dimer binding site prediction, a non-redundant set of eight TF dimer-DNA complex structures was used: 1 AM9, 1GU4, 1JNM, 1NKP, 1NLW, 1OZJ, 2QL2, and 2YPA.

### Performance evaluation

Due to the differences in length of predicted binding motifs and the heterogeneity of the TF domains that are involved in experimental determination of TFBSs, we calculated the *Information Content weighted Pearson Correlation Coefficient* (IC-weighted PCC) values and used the IC-weighted PCC values to determine the number of correctly predicted positions in the aligned PWMs between the predicted and reference motifs. IC-weighted PCC is a PWM comparison method developed by Persikov and Singh to measure the similarity of the corresponding columns between the predicted and the reference PWMs of the same base positions in the binding motif [47]. The information content was calculated using Eq. 4:

4 $$ IC(m)=2+{\sum}_{B\in \left\{ A, C, G, T\right\}}{m}_B \log \kern0.5em {m}_B $$

where the *IC*(*m*) is the information content function for column *m* in a PWM, and *B* represents DNA base frequencies in that column. The IC-weighted PCC was then calculated using Eq. 5:

5 $$ {PCC}_{m. n}^{IC}=\frac{\sum_{b\in \left\{ A, C, G, T\right\}}\left({m}_b-\overline{m}\right)\kern0.5em \left({n}_b-\overline{n}\right)}{\sqrt{\sum_{b\in \left\{ A, C, G, T\right\}}{\left({m}_b-\overline{m}\right)}^2\bullet {\sum}_{b\in \left\{ A, C, G, T\right\}}{\left({n}_b-\overline{n}\right)}^2}}\times \frac{IC(m)}{2} $$

where ${PCC}_{m, n}^{IC}$ is the IC-weighted PCC between the reference column *m*, and the predicted column *n*. *m*_*b*_ and *n*_*b*_ are the frequencies of the DNA bases *b*, found in the rows of the corresponding reference and predicted PWM columns respectively. $\overline{m}$ and $\overline{n}$ are the mean frequencies in the reference and predicted columns respectively. A predicted column is considered a correct prediction when the IC-weighted PCC between the corresponding predicted and reference columns is at least 0.25 [47]. The advantage of using IC-weighted PCC measure is that it takes into consideration both the conservation of a base-position in the reference binding motif (information content) and how well it matches the predicted binding motif (Pearson’s correlation coefficient). The statistical analysis was carried out using Wilcoxon Signed-rank test to compare the number of correctly predicted columns in a dataset.

Averaged Kullback-Liebler (AKL) divergence was also used to quantitatively measure the similarity between the predicted and reference PWMs as shown in Eq. 6 [48, 49].

6 $$ {D}_{A KL}={\sum}_i{\sum}_{B\in \left\{ A, C, T, G\right\}}\frac{\left({P}_{i B} log\frac{P_{i B}}{Q_{i B}}+{Q}_{i B} log\frac{Q_{i B}}{P_{i B}}\right)}{2} $$

where *D*_*AKL*_ is the AKL divergence, *i* represents the corresponding columns of the base positions being compared in the predicted and reference matrices. *B* represents the four bases A, C, G and T. *P*_*iB*_ and *Q*_*iB*_ represent the frequency of a particular base B in corresponding columns *i* in the predicted and reference matrices respectively.

## Results

In this work, in addition to the new pentamer algorithm, we proposed a simplified IE function for more efficient prediction over the previously developed IE function that requires an external program to calculate hydrogen bond energy (See Methods) [12]. To check if the new simplified IE energy function has comparable performance to the original IE function, we compared their prediction accuracy using the same prediction algorithm (termed *full-length algorithm* in this study). Wilcoxon Signed-rank test showed that there is no significant difference between the new IE and the original IE function when tested on the non-redundant dataset of 27 TF-DNA complex structures using the full-length prediction algorithm [12]. The *p-values* in terms of AKL divergence and the number of correctly predicted columns are 0.65 and 0.39 respectively for the null hypothesis: there is no difference between the two IE functions. For individual cases, one IE function may work better than the other but overall there is no apparent difference between the original IE function with explicit hydrogen bond and π-interaction energy terms and the new IE function even though the new IE function is much easier and less computationally intensive to calculate (Additional file 1: Figure S1).

**Fig. 4:**
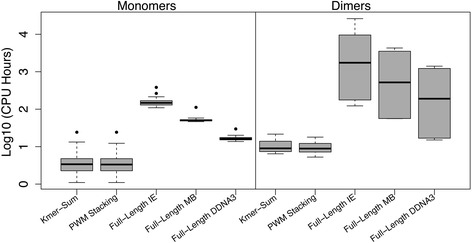
Comparison of CPU time on the non-redundant monomer and dimer sets with different prediction algorithms: Kmer-Sum, PWM stacking and full-length IE method, and with different energy functions: IE, MB, and DDNA3

The pentamer algorithms with the simplified IE function were tested on the multi-family non-redundant dataset of 27 TF monomer-DNA and 8 TF dimer-DNA complex structures. The performance of the new pentamer algorithm was compared with our previous full-length algorithm in terms of both speed and prediction accuracy. Computing time results on the monomer and the dimer cases clearly show that the pentamer algorithm is much faster than the full-length methods (Fig. 4 and Additional file 1: Table S2 for all the cases). In the TF monomer-DNA cases, the new algorithm requires an average of 4.66 (Kmer-Sum) and 4.61 (PWM stacking) CPU hours for IE energy calculation (columns 4 and 5 respectively in Additional file 1: Table S2) while the full-length IE method needs an average of 162 CPU hours (column 6 in Additional file 1: Table S2), about 35 times faster. Even though the pentamer algorithm requires a post-processing step to predict the binding sites by combining the pentamer energy in Kmer-Sum or PWM stacking (Fig. 3), the time for post-processing is minimal (data not shown). The DDNA3 and MB energy function in the full-length method required less time than the IE energy but they still used several fold (>3.7 for DDNA3 and >11 for MB) more time than the pentamer IE method (Additional file 1: Table S2) and they are less accurate than the IE function in TF binding site prediction as we demonstrated previously [12].

When tested on the dataset of TF dimers with longer DNA binding sites, the improvement in computing time is even more significant. The pentamer algorithm performs calculations at a linear time complexity for subsequences of the binding site, making prediction of longer binding motifs much faster (Additional file 1: Table S3). For example, the binding site for three TF dimers (1 AM9:human SREBP-1, 1GU4:human C/EBPβ, and 1OZJ:human Smad3-MH1) has 12 base pairs. The full-length algorithm needs to evaluate 4^12^ = 16,777,216 TF-DNA interaction energies while the pentamer algorithm only needs to calculate binding energy for 8*4^5^ = 8192 TF-pentamer complexes. Not surprisingly, the run-time (CPU hours) analysis showed that the pentamer algorithm, including TF-pentamer energy calculation and the Kmer-Sum post-processing time, has about 644 (for 1GU4) to 1209 (for 1OZJ) fold improvement over the full-length IE method (Fig. 4 and Additional file 1: Table S2). Another factor that affects the running time is the protein size (Additional file 1: Figure S2). It took longer for 1OZJ than that for 1GU4 since 1OZJ (288 amino acids) is about twice the size of 1GU4 (156 amino acids). Even though MB and DDNA3 energy calculations require less time than the IE energy, the pentamer algorithm still showed on average 143 times faster than full-length MB and 47.5 times faster than full-length DDNA3.

**Fig. 5:**
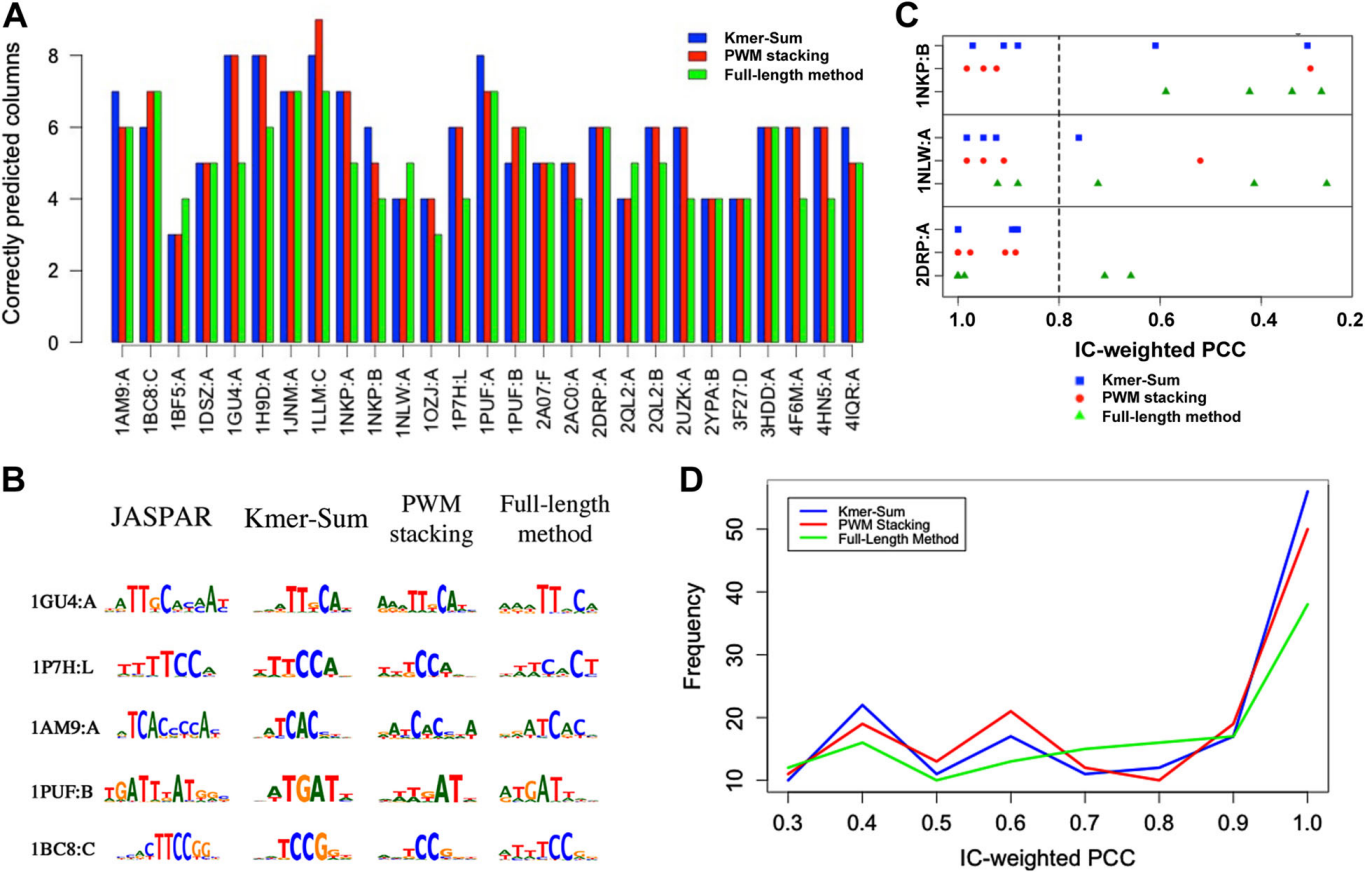
Comparison of TF binding site prediction. **a** Comparison of the number of correctly predicted columns (based on the IC-weighted PCC scores) by the Kmer-Sum (*blue*), PWM stacking (*red*), and previous full-length (*green*) algorithms. **b** Examples of five binding motifs predicted using different methods. **c** Examples of distributions of IC-weighted PCC values of correctly predicted columns by Kmer-Sum (*blue squares*), PWM stacking (*red circles*), and full-length (*green triangles*) algorithms. **d** Binned distributions of IC-weighted PCC scores (≥0.25) in 27 cases by the Kmer-Sum (*blue*), PWM stacking (*red*), and full-length (*green*) algorithms in the multi-family dataset

**Fig. 6:**
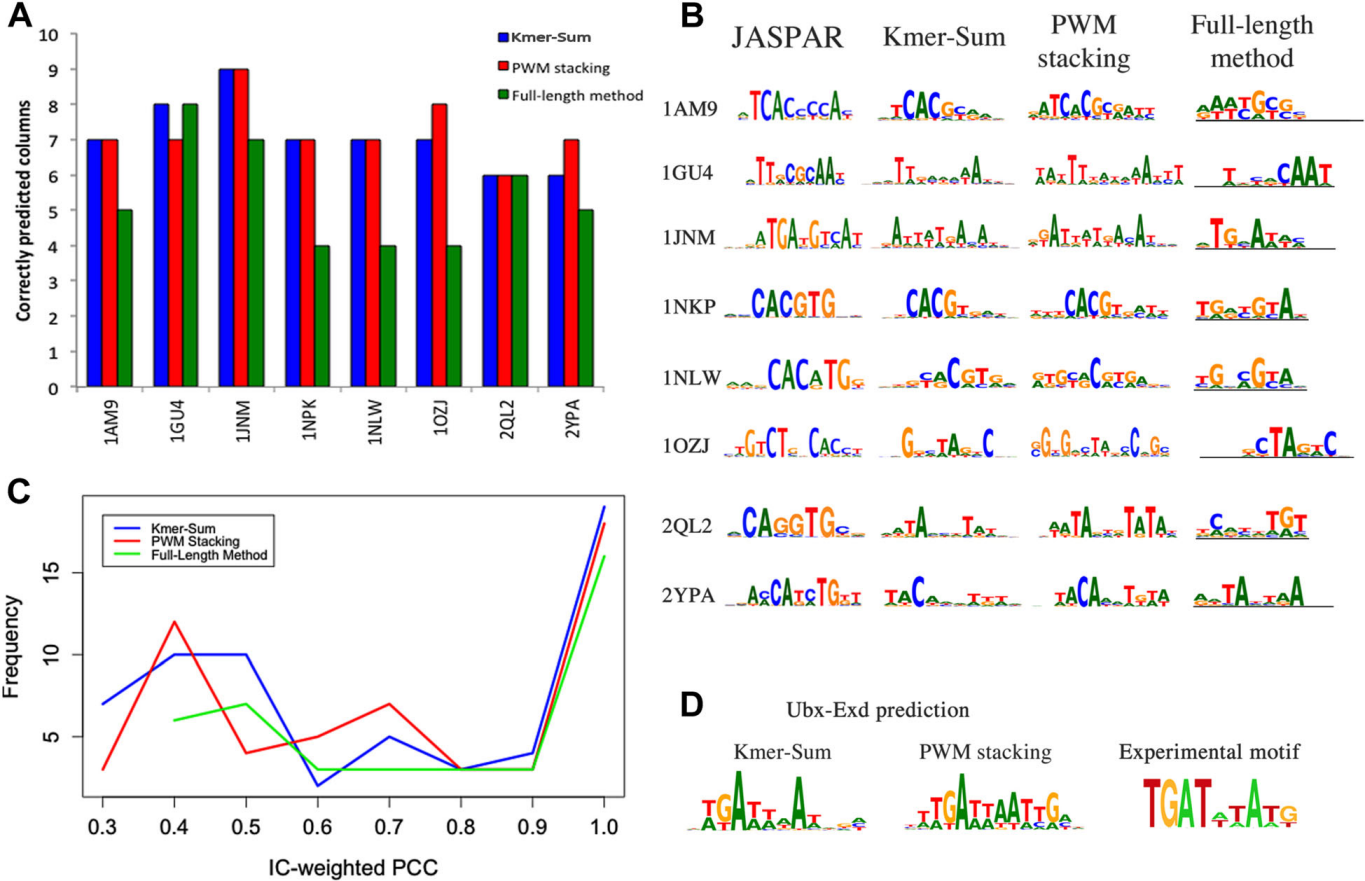
Prediction of TF dimer binding sites. **a** Comparison of the number of correctly predicted columns in TF dimers by the Kmer-Sum (*blue*), PWM stacking (*red*), and full-length (*green*) algorithms. **b** Comparison of TF binding motifs of TF dimers using the Kmer-Sum, PWM stacking, and full-length algorithms. **c** Distribution of the IC-weighted PCC values above 0.25 from three different prediction algorithms. **d** Multi-domain TF prediction of the Ubx-Exd TFBS

Not only does the pentamer algorithm run much faster due to the reduced total number of energy calculations, it also produced overall better results than the original full-length method in terms of the number of correctly predicted columns (Figs. 5 and 6). For example, more columns are predicted correctly using either the Kmer-Sum or the PWM stacking pentamer algorithm for 1GU4:A and 1P7H:L (human NFAT1), which are also reflected in the binding motifs (Fig. 5a and b). (See Additional file 1: Figure S3 for all 27 predicted TF monomer binding motifs). We performed statistical analysis using Wilcoxon Signed-rank test to test the alternative hypothesis that the pentamer algorithm generated a greater number of correctly predicted base positions than the previous algorithm. The *p*-values are 0.0028 and 0.0029 for the Kmer-Sum and PWM stacking algorithms respectively, suggesting that increases in prediction accuracy are statistically significant for both the Kmer-Sum and the PWM stacking methods over the full-length method using the IE function.

In several cases, even though both the pentamer and the full-length methods show similar results in terms of the number of correctly predicted columns, the results from the pentamer algorithm are actually better than the previous method because of the broad definition of correctly predicted columns. The cutoff for correctly predicted columns is set at 0.25 (IC-weighted PCC value) as proposed by Persikov and Singh [47], meaning there is a large range, from 0.25 to 1, of IC-weighted PCC values for the correctly predicted columns. A closer look at the distributions of the IC-weighted PCC values revealed that even though the correctly predicted columns are comparable between the pentamer and the full-length algorithms, including 2A07:F (human Foxp2), 1NLW:A (human MAD protein), and 2DRP:A (*Drosophila melanogaster* Cys2-His2 zinc finger), the actual IC-weighted PCC values from the pentamer predictions are better (closer to 1) than the IC-weighted PCC values from the previous method (Fig. 5a and c). The distributions of the IC-weighted PCC of all 27 cases shows a similar trend; more data points are close to the perfect IC-weighted PCC score in the pentamer algorithms than the full-length method (Fig. 5d and Additional file 1: Figure S4).

While the pentamer method significantly reduces the time for energy calculation for longer binding sites (Fig. 4 and Additional file 1: Table S2), more importantly, we found that it also improved the prediction accuracy significantly. Six of the eight dimer cases showed much improved TFBS predictions when compared to predictions using the full-length method (Fig. 6a-c and Additional file 1: Figure S5). Wilcoxon Signed-rank tests were performed to test the alternative hypothesis that the pentamer algorithms have more correctly predicted columns, which showed the differences are statistically significant with *p-values* of 0.0168 and 0.0165 for the Kmer-Sum and PWM stacking pentamer algorithms respectively.

In addition to TF dimers with known TF-DNA complex structures and corresponding JASPAR motifs, we also made predictions for Hox proteins Extradenticle (Exd) and Ultrabithorax (Ubx). Ubx and Exd form a dimer to regulate gene expression [50, 51]. Though both Ubx and Exd have annotated JASPAR PWMs separately, there are no JASPAR binding motifs for the Ubx-Exd dimer. However, binding sites of Ubx-Exd dimer have been reported in several studies [50, 52]. The predicted Ubx-Exd dimer (PDB ID: 1B8I) binding sites are consistent with the published data (Fig. 6d). Furthermore, the *Drosophila* limb-promoting gene *Distalless* regulatory element, which is in part regulated by Ubx-Exd interactions, also validates the pentamer prediction results [53, 54].

## Discussion

We have previously developed a structure-based method using an IE function for improving TF binding site prediction and demonstrated that it increased prediction accuracy for different TF families as well as different TFs within the homeodomain family [12]. The method needed to evaluate *n* ^*L*^ TF-DNA complex structures (where *L* is the length of the binding sequence). Since a fixed sequence length (8 bps) was used for TF binding site prediction for each single TF domain-DNA complex, each prediction required energy calculations for all 65,536 (4^8^) possible permutations of the sequence, which could be calculated in a reasonable time frame [12]. However, as the length of the binding motif increases, the number of energy calculations increases exponentially, making it impractical for longer TF binding site predictions even with the availability of large computer clusters. There are many instances where we need to evaluate longer binding sequences. For example, we need to consider flanking sequences for binding site prediction as it has been demonstrated that flanking bases contribute to binding specificity even though these flanking sequences are not conserved [42]. Secondly, some binding sites are regulated by dimers or tetramers of either the same transcription factor (homo-) or different transcription factors (hetero-), which are typically much longer than 8 base pairs. Also in homology model based TF binding site prediction, it would be ideal to consider multiple homology models to increase the conformational coverage, which demands more energy calculations.

We addressed the problem in this study by developing a simplified IE function and a fragment-based pentamer algorithm, which improve both the speed and prediction accuracy, especially for longer binding sites (Figs. 4, 5 and 6 and Additional file 1: Table S2). The increase of prediction speed is not surprising since we only need to calculate energies of 1024 (4^5^) TF-pentamer complexes times the number of fragments (Fig. 2 and Additional file 1: Table S3). The overall improvement of accuracy may lie in the fact that the long-range interactions from the coarse multibody function can introduce noise to our previous full-length algorithm. In the pentamer algorithm the noise level is reduced since it only considers a short sequence environment. Another factor that may affect the prediction accuracy is the weights for the two energy terms in the simplified IE function. As mentioned in the Methods section, training for an optimal set of weights is not possible due to the limited availability of cases. The weights, 1 for MB term and 0.5 for the electrostatic energy term, were assigned based on our previous study and the characteristics of the energy terms [12]. For example, knowledge-based multibody potential already implicitly captured the electrostatic interactions that are important for specific protein-DNA interactions, including hydrogen bonds and π-interactions. To investigate the effects of weights on the prediction performance, we compared the prediction accuracy of different weight ratios. Statistical analyses showed that the weight combination in our study was among a small number of combinations that produced better predictions and the weights for the electrostatic energy term *W*_*E*_ are between 0.25 (4^−1^) to 0.5 (2^−1^) (Additional file 1: Figure S6).

As a note, while the pentamer algorithm reduces the time complexity by lowering the number of TF-DNA complexes for energy calculation, calculating the final binding motif using the Kmer-Sum algorithm requires additional computing time especially for longer binding sites as it requires calculation for all possible sequence permutations for the full length binding site (Fig. 3a). Nevertheless, the total time used by the pentamer algorithm is significantly less than the full-length method with better prediction accuracy. For dimer structures and multi-domain TFs that have longer spacing (> = 4 bps) between two monomer binding sites, it may be more efficient to calculate each of the binding sites individually and then combine them to form one binding motif. Of the two pentamer combination algorithms, PWM stacking is marginally faster as it does not need to calculate the individual sequence binding energy while the Kmer-Sum algorithm has slightly better prediction accuracy. In addition, the Kmer-Sum method actually predicts energy scores for each binding sequences while PWM stacking can only produce a binding sequence profile.

## Conclusion

We developed a fragment-based pentamer algorithm with a simplified energy function that greatly speeds up the TFBS prediction by reducing the number of energy calculations and improves TFBS prediction accuracy. Two algorithms, Kmer-Sum and PWM stacking, were used to combine the TF-pentamer scores for binding motif prediction with overall better performance in terms of TFBS prediction accuracy. Our results also show that the longer the binding sites, the more speedup and accuracy can be achieved. For future studies, we will test this new approach for binding site prediction using TF-DNA homology models.

## Additional file

Additional file 1: Supplemental figures and tables. (PDF 3646 kb)

## Abbreviations

AKL: Average Kullback-Liebler
Exd: Extradenticle
HB: Hydrogen bond
IC: Information content
IE: Integrative energy
MB: Multibody
PCC: Pearson’s correlation coefficient
PDB: Protein Data Bank
PFM: Position frequency matrix
PWM: Position weight matrix
RMSD: Root mean squared deviations
TF: Transcription factor
TFBS: Transcription factor binding site
Ubx: Ultrabithorax
VDW: Van der Waals

## References

[1] Lemon B, Tjian R (2000). Orchestrated response: a symphony of transcription factors for gene control. Genes Dev.

[2] Levine M, Tjian R (2003). Transcription regulation and animal diversity. Nature.

[3] Guo J-T, Lofgren S, Farrel A (2014). Structure-based prediction of transcription factor binding sites. Tsinghua Sci Technol.

[4] Liu LA, Bradley P (2012). Atomistic modeling of protein-DNA interaction specificity: progress and applications. Curr Opin Struct Biol.

[5] Berman HM, Bhat TN, Bourne PE, Feng ZK, Gilliland G, Weissig H, Westbrook J (2000). The protein data Bank and the challenge of structural genomics. Nat Struct Biol.

[6] Endres RG, Schulthess TC, Wingreen NS (2004). Toward an atomistic model for predicting transcription-factor binding sites. Proteins.

[7] Kono H, Sarai A (1999). Structure-based prediction of DNA target sites by regulatory proteins. Proteins.

[8] Morozov AV, Havranek JJ, Baker D, Siggia ED (2005). Protein-DNA binding specificity predictions with structural models. Nucleic Acids Res.

[9] Garvie CW, Wolberger C (2001). Recognition of specific DNA sequences. Mol Cell.

[10] Kaplan T, Friedman N, Margalit H (2005). Ab initio prediction of transcription factor targets using structural knowledge. PLoS Comput Biol.

[11] Siggers TW, Honig B (2007). Structure-based prediction of C2H2 zinc-finger binding specificity: sensitivity to docking geometry. Nucleic Acids Res.

[12] Farrel A, Murphy J, Guo JT (2016). Structure-based prediction of transcription factor binding specificity using an integrative energy function. Bioinformatics.

[13] Mandel-Gutfreund Y, Margalit H (1998). Quantitative parameters for amino acid-base interaction: implications for prediction of protein-DNA binding sites. Nucleic Acids Res.

[14] Aloy P, Moont G, Gabb HA, Querol E, Aviles FX, Sternberg MJ (1998). Modelling repressor proteins docking to DNA. Proteins.

[15] Liu Z, Mao F, Guo JT, Yan B, Wang P, Qu Y, Xu Y (2005). Quantitative evaluation of protein-DNA interactions using an optimized knowledge-based potential. Nucleic Acids Res.

[16] Takeda T, Corona RI, Guo JT (2013). A knowledge-based orientation potential for transcription factor-DNA docking. Bioinformatics.

[17] Donald JE, Chen WW, Shakhnovich EI (2007). Energetics of protein-DNA interactions. Nucleic Acids Res.

[18] Zhang C, Liu S, Zhu Q, Zhou Y (2005). A knowledge-based energy function for protein-ligand, protein-protein, and protein-DNA complexes. J Med Chem.

[19] Robertson TA, Varani G (2007). An all-atom, distance-dependent scoring function for the prediction of protein-DNA interactions from structure. Proteins.

[20] Xu B, Yang Y, Liang H, Zhou Y (2009). An all-atom knowledge-based energy function for protein-DNA threading, docking decoy discrimination, and prediction of transcription-factor binding profiles. Proteins.

[21] Lu XJ, Olson WK (2003). 3DNA: a software package for the analysis, rebuilding and visualization of three-dimensional nucleic acid structures. Nucleic Acids Res.

[22] Lu XJ, Olson WK (2008). 3DNA: a versatile, integrated software system for the analysis, rebuilding and visualization of three-dimensional nucleic-acid structures. Nat Protoc.

[23] Simons KT, Kooperberg C, Huang E, Baker D (1997). Assembly of protein tertiary structures from fragments with similar local sequences using simulated annealing and Bayesian scoring functions. J Mol Biol.

[24] Zhang Y (2007). Template-based modeling and free modeling by I-TASSER in CASP7. Proteins.

[25] Chiu TP, Yang L, Zhou T, Main BJ, Parker SC, Nuzhdin SV, Tullius TD, Rohs R (2015). GBshape: a genome browser database for DNA shape annotations. Nucleic Acids Res.

[26] Yang L, Orenstein Y, Jolma A, Yin Y, Taipale J, Shamir R, Rohs R (2017). Transcription factor family-specific DNA shape readout revealed by quantitative specificity models. Mol Syst Biol.

[27] Zhou T, Shen N, Yang L, Abe N, Horton J, Mann RS, Bussemaker HJ, Gordan R, Rohs R (2015). Quantitative modeling of transcription factor binding specificities using DNA shape. Proc Natl Acad Sci U S A.

[28] Zhou T, Yang L, Lu Y, Dror I, Dantas Machado AC, Ghane T, Di Felice R, Rohs R (2013). DNAshape: a method for the high-throughput prediction of DNA structural features on a genomic scale. Nucleic Acids Res.

[29] Jacobs DJ, Rader AJ, Kuhn LA, Thorpe MF (2001). Protein flexibility predictions using graph theory. Proteins.

[30] ChemAxon [http://www.chemaxon.com]. Accessed July 2017.

[31] Thorpe MF, Lei M, Rader AJ, Jacobs DJ, Kuhn LA (2001). Protein flexibility and dynamics using constraint theory. J Mol Graph Model.

[32] Dahiyat BI, Mayo SL (1997). De novo protein design: fully automated sequence selection. Science.

[33] Pettersen EF, Goddard TD, Huang CC, Couch GS, Greenblatt DM, Meng EC, Ferrin TE (2004). UCSF chimera--a visualization system for exploratory research and analysis. J Comput Chem.

[34] Word JM, Lovell SC, Richardson JS, Richardson DC (1999). Asparagine and glutamine: using hydrogen atom contacts in the choice of side-chain amide orientation. J Mol Biol.

[35] Gallivan JP, Dougherty DA (1999). Cation-pi interactions in structural biology. Proc Natl Acad Sci U S A.

[36] Michael Gromiha M, Siebers JG, Selvaraj S, Kono H, Sarai A (2004). Intermolecular and intramolecular readout mechanisms in protein-DNA recognition. J Mol Biol.

[37] McGaughey GB, Gagne M, Rappe AK (1998). Pi-stacking interactions. Alive and well in proteins. J Biol Chem.

[38] Wintjens R, Lievin J, Rooman M, Buisine E (2000). Contribution of cation-pi interactions to the stability of protein-DNA complexes. J Mol Biol.

[39] SantaLucia J, Allawi HT, Seneviratne PA (1996). Improved nearest-neighbor parameters for predicting DNA duplex stability. Biochemistry.

[40] Afek A, Schipper JL, Horton J, Gordan R, Lukatsky DB (2014). Protein-DNA binding in the absence of specific base-pair recognition. Proc Natl Acad Sci U S A.

[41] Barrera LA, Vedenko A, Kurland JV, Rogers JM, Gisselbrecht SS, Rossin EJ, Woodard J, Mariani L, Kock KH, Inukai S (2016). Survey of variation in human transcription factors reveals prevalent DNA binding changes. Science.

[42] Gordan R, Shen N, Dror I, Zhou T, Horton J, Rohs R, Bulyk ML (2013). Genomic regions flanking E-box binding sites influence DNA binding specificity of bHLH transcription factors through DNA shape. Cell Rep.

[43] Slattery M, Zhou T, Yang L, Dantas Machado AC, Gordan R, Rohs R (2014). Absence of a simple code: how transcription factors read the genome. Trends Biochem Sci.

[44] Schneider TD, Stephens RM (1990). Sequence logos: a new way to display consensus sequences. Nucleic Acids Res.

[45] Crooks GE, Hon G, Chandonia JM, Brenner SE (2004). WebLogo: a sequence logo generator. Genome Res.

[46] Mathelier A, Fornes O, Arenillas DJ, Chen CY, Denay G, Lee J, Shi W, Shyr C, Tan G, Worsley-Hunt R (2016). JASPAR 2016: a major expansion and update of the open-access database of transcription factor binding profiles. Nucleic Acids Res.

[47] Persikov AV, Singh M (2014). De novo prediction of DNA-binding specificities for Cys2His2 zinc finger proteins. Nucleic Acids Res.

[48] Wu TJ, Hsieh YC, Li LA (2001). Statistical measures of DNA sequence dissimilarity under Markov chain models of base composition. Biometrics.

[49] Xu M, Su Z (2010). A novel alignment-free method for comparing transcription factor binding site motifs. PLoS One.

[50] Passner JM, Ryoo HD, Shen L, Mann RS, Aggarwal AK (1999). Structure of a DNA-bound Ultrabithorax-Extradenticle homeodomain complex. Nature.

[51] Crocker J, Abe N, Rinaldi L, McGregor AP, Frankel N, Wang S, Alsawadi A, Valenti P, Plaza S, Payre F (2015). Low affinity binding site clusters confer hox specificity and regulatory robustness. Cell.

[52] Foos N, Maurel-Zaffran C, Mate MJ, Vincentelli R, Hainaut M, Berenger H, Pradel J, Saurin AJ, Ortiz-Lombardia M, Graba Y (2015). A flexible extension of the drosophila ultrabithorax homeodomain defines a novel Hox/PBC interaction mode. Structure.

[53] Gebelein B, Culi J, Ryoo HD, Zhang W, Mann RS (2002). Specificity of Distalless repression and limb primordia development by abdominal Hox proteins. Dev Cell.

[54] Merabet S, Saadaoui M, Sambrani N, Hudry B, Pradel J, Affolter M, Graba Y (2007). A unique Extradenticle recruitment mode in the drosophila Hox protein Ultrabithorax. Proc Natl Acad Sci U S A.

