# Supplementary material for: An efficient algorithm for improving structure-based prediction of transcription factor binding sites
Source: BMC Bioinformatics. 2017 Jul 17;18:342. doi: 10.1186/s12859-017-1755-0 (PMC5514533; doi:10.1186/s12859-017-1755-0)

Table S1: Charge fractions of atoms for calculation of electrostatic potential

Amino Acid	Atom	Charge fraction	Amino Acid	Atom	Charge fraction
SER	HG	0.21	PHE	CZ	-0.372
	HB2	0.056		CG	-0.372
	HB3	0.056		HD1	0.062
	OG	-0.397		HD2	0.062
THR	HG1	0.21		HE1	0.062
	HB	0.059		HE2	0.062
	OG1	-0.394		HZ	0.062
ASP	OD1	-0.482	TRP	CG	-0.685
	OD2	-0.482		CH2	-0.685
GLU	OE1	-0.482		HD1	0.104
	OE2	-0.482		HE1	0.252
ASN	OD1	-0.299		HZ2	0.054
	D21	0.127	DG	N3	-0.206
	D22	0.127		H21	0.152
GLN	OE1	-0.298		H22	0.152
	E21	0.127		H1	0.174
	E22	0.127		O6	-0.441
ARG	HE	0.140		N7	-0.215
	H11	0.268		H8	0.107
	H12	0.268	DA	N3	-0.239
	H21	0.292		N1	-0.241
	H22	0.292		H2	0.089
LYS	HZ1	0.255		H61	0.157
	HZ2	0.255		H62	0.157
	HZ3	0.255		N7	-0.21
TYR	CZ	-0.249		H8	0.115
	CG	-0.249	DC	O2	-0.468
	HD1	0.062		N3	-0.22
	HD2	0.062		H41	0.157
	HE1	0.065		H42	0.157
	HE2	0.065		H5	0.066
	HH	0.218		H6	0.085
CYS	HG	0.102	DT	O2	-0.373
HIS	ND1	-0.255		H3	0.194
	HD2	0.068		O4	-0.478
	HE1	0.107		H6	0.096
	HE2	0.217			

Table S2: Running time (CPU hours) comparison between the pentamer algorithms and the full-length algorithm with different energy functions (IE, MB, and DDNA3).

PDB ID	Structure Type	TFBS Length	Pentamer (Kmer-Sum) CPU Hours	Pentamer PWM Stacking CPU Hours	Full Length IE CPU Hours	Full Length MB CPU Hours	Full Length DDNA3 CPU Hours
**1AM9**	Dimer	12mer	7.05	6.85	8843.57	3676.47	1316.76
**1GU4**	Dimer	12mer	16.07	12.54	10350.79	3380.99	1136.47
**1OZJ**	Dimer	12mer	21.60	17.96	26125.9	4300.86	1408.2
**1JNM**	Dimer	10mer	17.96	11.88	1736.43	979.28	279.72
**2YPA**	Dimer	10mer	9.27	9.04	4101.66	1110.06	327.79
**1NKP**	Dimer	8mer	8.93	8.73	187.04	56.7	16.56
**1NLW**	Dimer	8mer	7.72	7.52	166.14	56.01	17.13
**2QL2**	Dimer	8mer	6.44	5.26	122.62	55.97	15.09
**1AM9:A**	Monomer	8mer	3.25	3.25	263.93	53.89	16.2
**1BC8:C**	Monomer	8mer	2.15	2.15	109.29	47.57	14.82
**1BF5:A**	Monomer	8mer	24.27	24.27	382.78	112	29.71
**1DSZ:A**	Monomer	8mer	3.39	3.33	129.27	49.02	15.35
**1GU4:A**	Monomer	8mer	4.17	4.16	116.75	47.67	15.45
**1H9D:A**	Monomer	8mer	3.88	3.87	129.87	50.67	15.45
**1JNM:A**	Monomer	8mer	5.43	5.39	118.16	48.47	15.09
**1LLM:C**	Monomer	8mer	3.29	3.28	167.82	46.55	15.07
**1NKP:A**	Monomer	8mer	2.41	2.41	149.04	50.22	16.73
**1NKP:B**	Monomer	8mer	2.40	2.4	136.04	51.08	18.53
**1NLW:A**	Monomer	8mer	2.08	2.08	139.01	52.04	17.7
**1OZJ:A**	Monomer	8mer	7.04	7.03	205.11	54.19	18.8
**1P7H:L**	Monomer	8mer	6.03	6.03	214.49	57.86	17.7
**1PUF:A**	Monomer	8mer	3.64	3.64	141.25	50.65	16.18
**1PUF:B**	Monomer	8mer	3.98	3.98	149.61	49.6	17.35
**2A07:F**	Monomer	8mer	3.13	3.13	126.37	49.23	15.32
**2AC0:A**	Monomer	8mer	5.26	5.26	169.91	58.09	18.79
**2DRP:A**	Monomer	8mer	7.28	6.99	152.71	50.38	20.14
**2QL2:A**	Monomer	8mer	1.82	1.82	112.71	50.46	17.25
**2QL2:B**	Monomer	8mer	1.94	1.94	119.96	50.53	16.88
**2UZK:A**	Monomer	8mer	4.36	4.35	162.52	51.35	15.28
**2YPA:B**	Monomer	8mer	2.02	2.02	147.57	48.1	13.77
**3HDD:A**	Monomer	8mer	4.25	4.24	142.43	57.32	16.56
**3F27:D**	Monomer	8mer	1.10	1.1	129.6	48.53	14.86
**4F6M:A**	Monomer	8mer	13.28	12.28	160.86	53.03	16.48
**4HN5:A**	Monomer	8mer	1.55	1.54	194.74	48.16	13.7
**4IQR:A**	Monomer	8mer	2.55	2.55	205.71	47.71	18.03

Table S3: The number of energy calculations for the full-length and pentamer algorithms.

DNA sequence length L (bp)	# of energy calculations in full-length method (4^L)	# of energy calculations in pentamer method (L-4)*1024
8	65,536	4,096
9	262,144	5,120
10	1,048,576	6,144
11	4,194,304	7,168
12	16,777,216	8,192
13	67,108,864	9,216
14	268,435,456	10,240
15	1,073,741,824	11,264

Figure S1: Comparison of prediction accuracy between the original IE and the modified IE energy functions. The predictions were carried out using the same full-length prediction algorithm. The prediction accuracy is presented using the AKL values calculated between the predicted binding motifs and the JASPAR reference motifs.

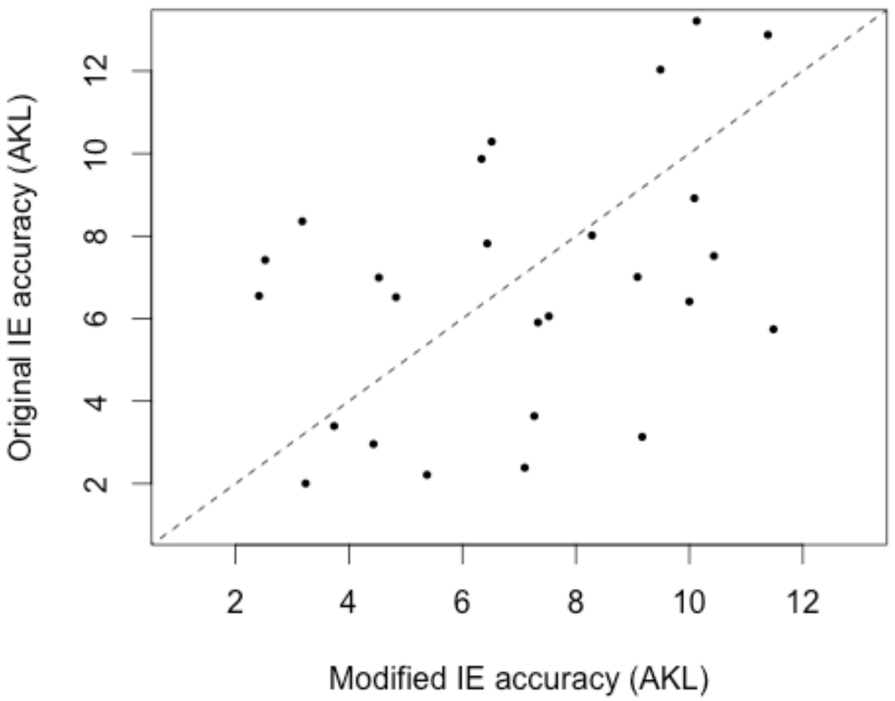

Figure S2: CPU time (CPU hours) vs. protein size (AAs: the number of amino acids) on the monomer dataset (A and B) and the dimer set (C and D) with Kmer-Sum Pentamer method (A and C) or Full-length IE method (B and D).

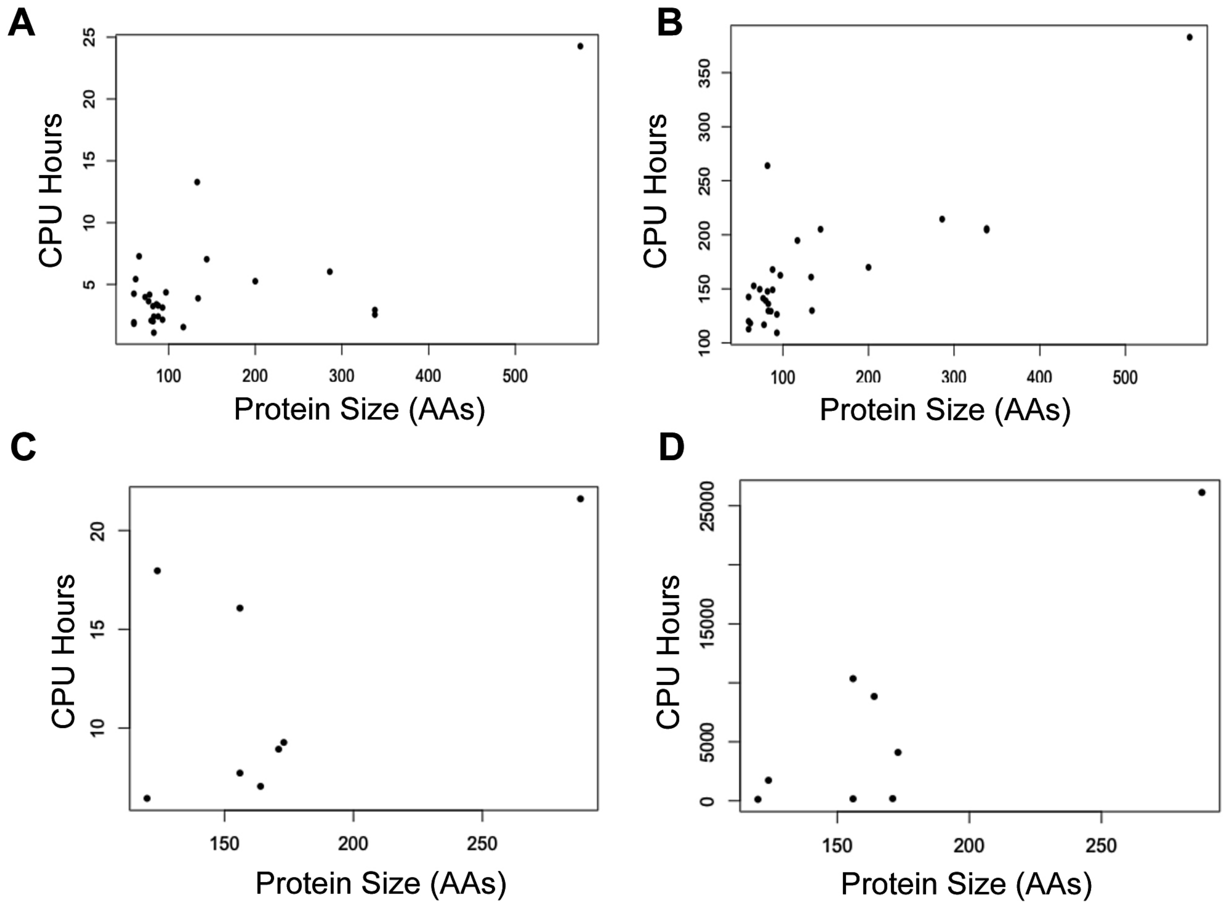

Figure S3: Comparison of the predicted motifs with the reference binding motifs in JASPAR for 27 TF Chain-DNA complexes in the non-redundant dataset.

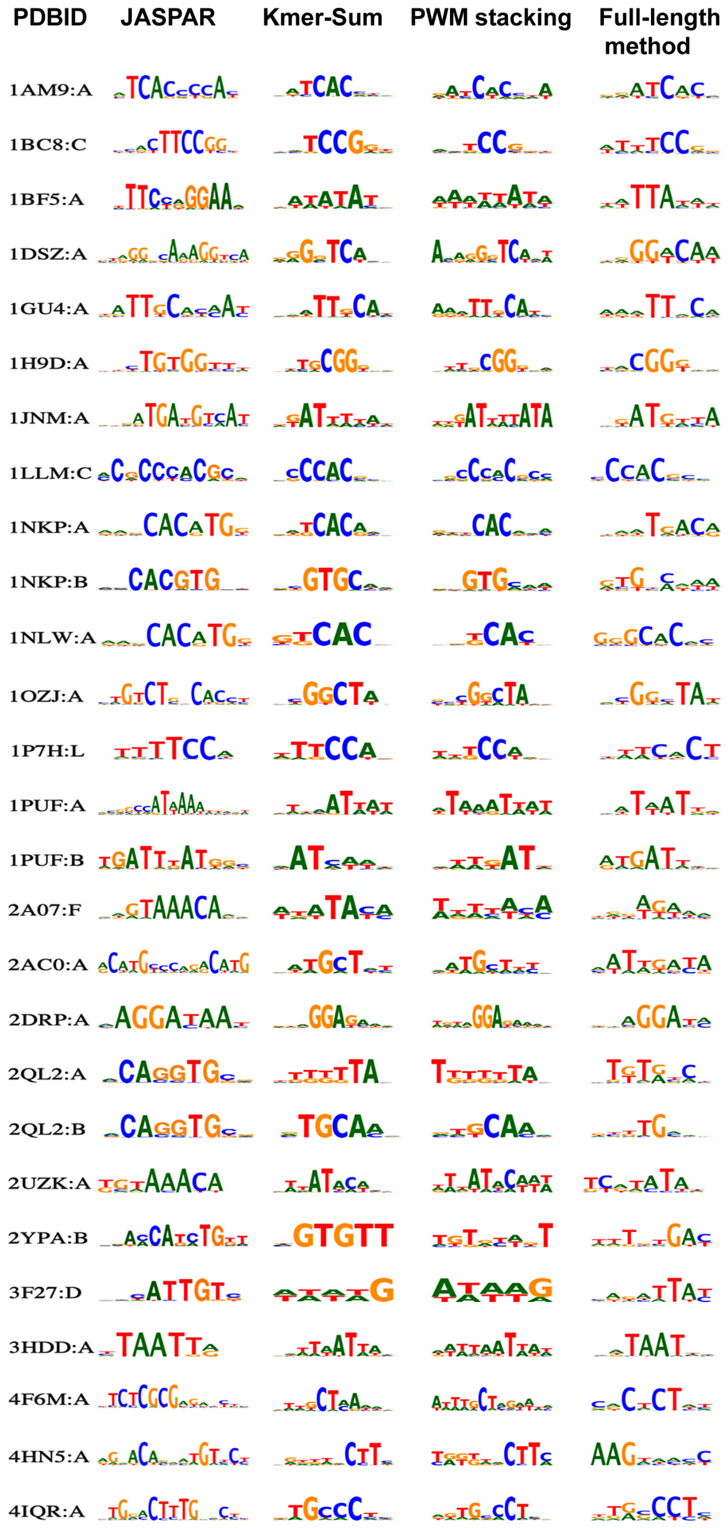

Figure S4: Distributions of IC-weighted PCC values of correctly predicted columns by Kmer-Sum (blue squares), PWM stacking (red circles), and full-length (green triangles) algorithms on the non-redundant dataset of 27 TF Chain-DNA complexes.

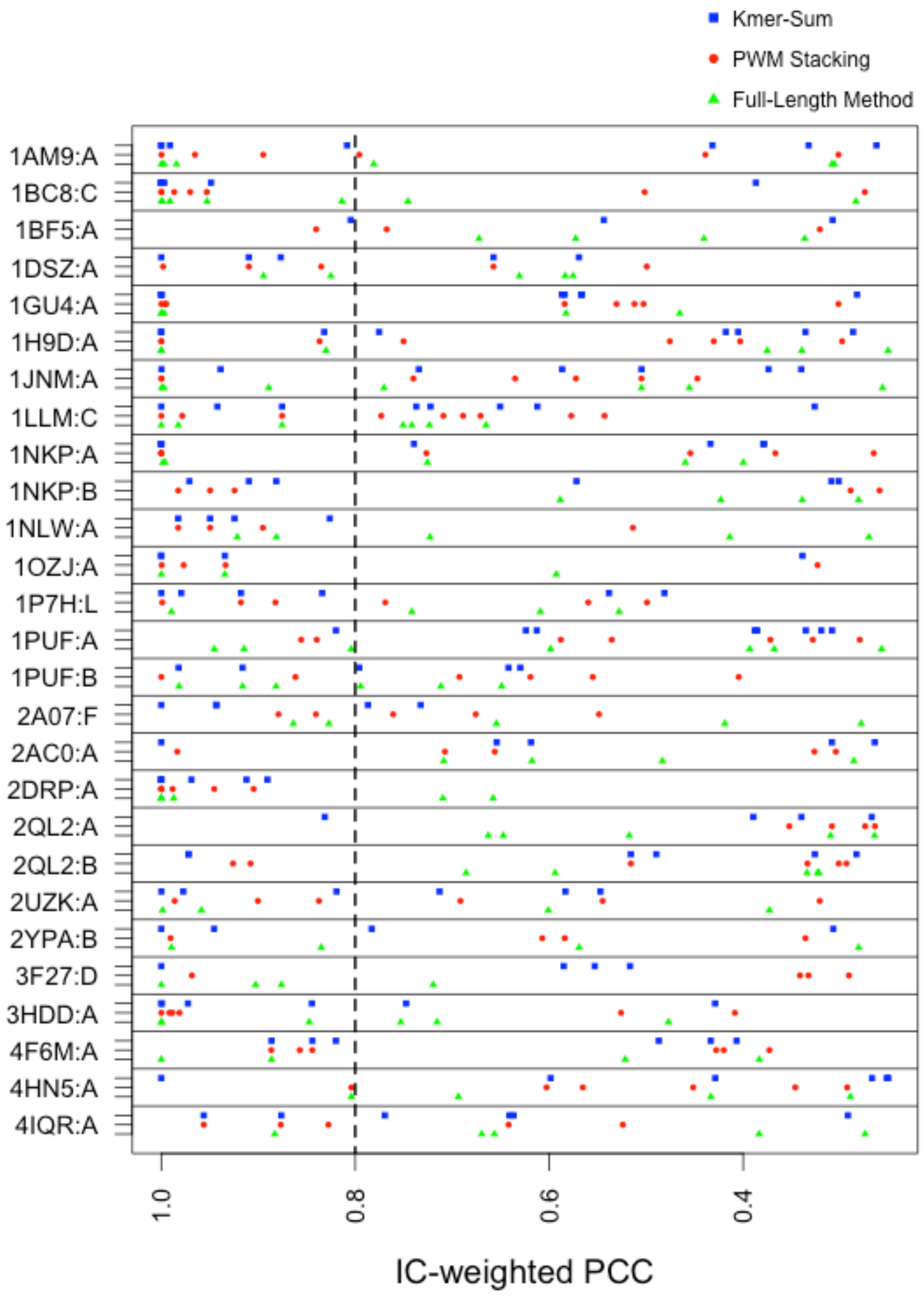

Figure S5: Distributions of IC-weighted PCC values of correctly predicted columns by Kmer-Sum (blue squares), PWM stacking (red circles), and full-length (green triangles) algorithms on the non-redundant dimer dataset of TF-DNA complexes.

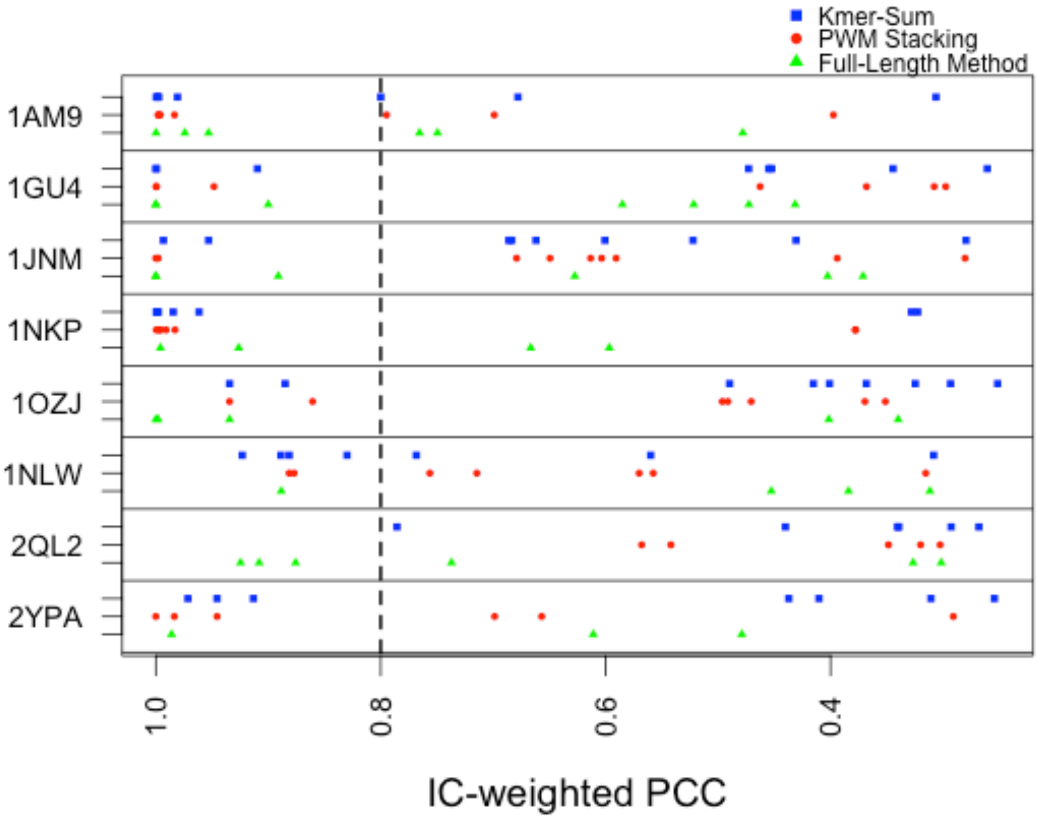

Figure S6: Performance comparison of different weight ratios between the electrostatic term W_E and the knowledge-based multibody potential W_{MB}. The *p-value* heatmap was generated from Wilcoxon signed-rank tests with an alternative hypothesis: TF-binding sites predicted with the electrostatic weight W_E in the rows/y-axis have greater number of correctly predicted columns than those predicted by the electrostatic weight W_E in the columns/x-axis when W_{MB} was fixed at 1. The green arrow indicates the weight W_E used in all the predictions in the paper. The color range from blue to red corresponds to *p-values* from 0 to 1.

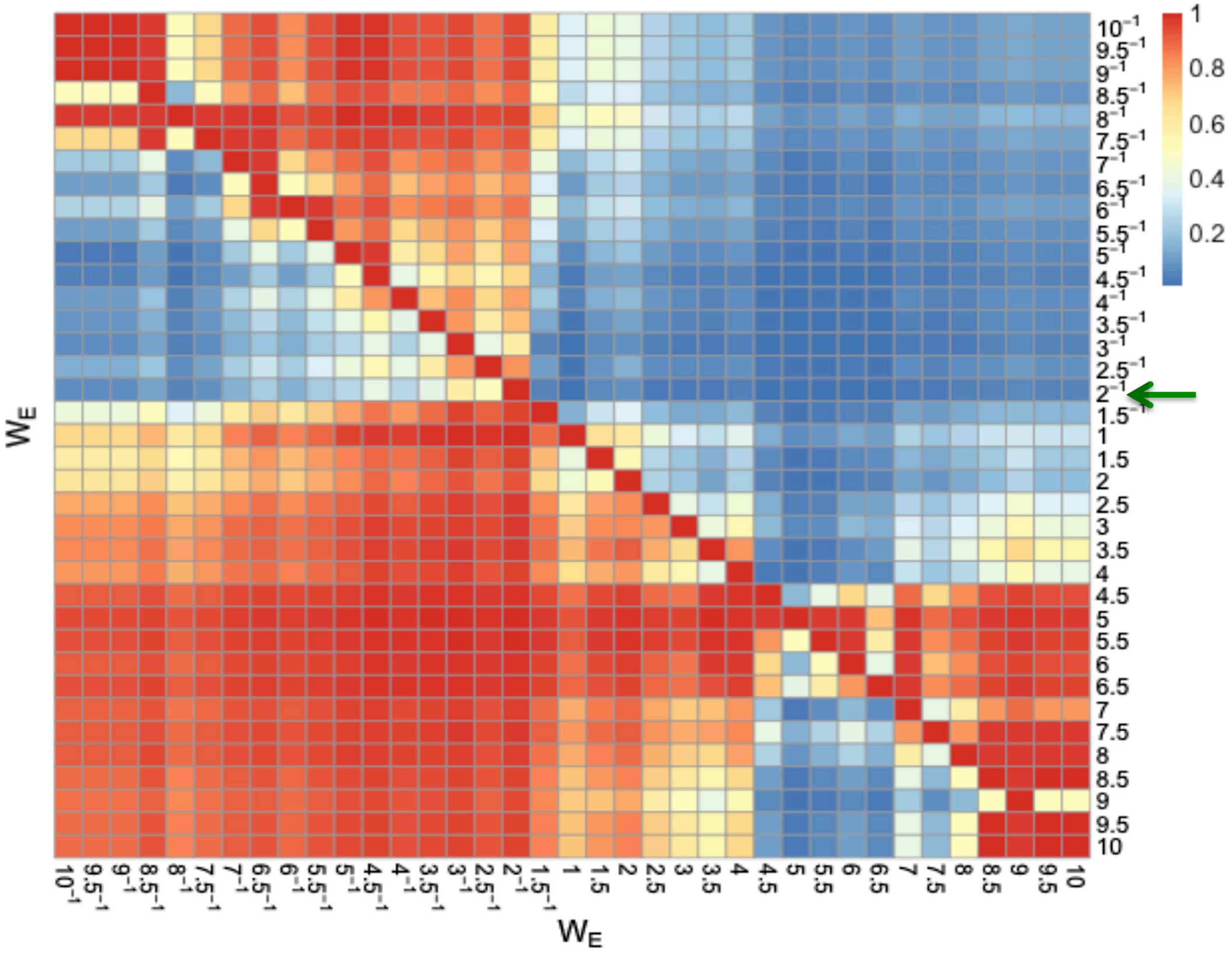

Supplement: Additional file 1: — Supplemental figures and tables. (PDF 3646 kb) [file 12859_2017_1755_MOESM1_ESM.pdf]
